# Expansion of Cord Blood CD34^+^ Cells in Presence of zVADfmk and zLLYfmk Improved Their In Vitro Functionality and In Vivo Engraftment in NOD/SCID Mouse

**DOI:** 10.1371/journal.pone.0012221

**Published:** 2010-08-17

**Authors:** Sangeetha V. M, Vaijayanti P. Kale, Lalita S. Limaye

**Affiliations:** Stem Cell Biology Laboratory, National Centre for Cell Science, Pune, Maharashtra, India; Cleveland Clinic, United States of America

## Abstract

**Background:**

Cord blood (CB) is a promising source for hematopoietic stem cell transplantations. The limitation of cell dose associated with this source has prompted the ex vivo expansion of hematopoietic stem and progenitor cells (HSPCs). However, the expansion procedure is known to exhaust the stem cell pool causing cellular defects that promote apoptosis and disrupt homing to the bone marrow. The role of apoptotic machinery in the regulation of stem cell compartment has been speculated in mouse hematopoietic and embryonic systems. We have consistently observed an increase in apoptosis in the cord blood derived CD34^+^ cells cultured with cytokines compared to their freshly isolated counterpart. The present study was undertaken to assess whether pharmacological inhibition of apoptosis could improve the outcome of expansion.

**Methodology/Principal Findings:**

CB CD34^+^ cells were expanded with cytokines in the presence or absence of cell permeable inhibitors of caspases and calpains; zVADfmk and zLLYfmk respectively. A novel role of apoptotic protease inhibitors was observed in increasing the CD34^+^ cell content of the graft during ex vivo expansion. This was further reflected in improved in vitro functional aspects of the HSPCs; a higher clonogenicity and long term culture initiating potential. These cells sustained superior long term engraftment and an efficient regeneration of major lympho-myeloid lineages in the bone marrow of NOD/SCID mouse compared to the cells expanded with growth factors alone.

**Conclusion/Significance:**

Our data show that, use of either zVADfmk or zLLYfmk in the culture medium improves expansion of CD34^+^ cells. The strategy protects stem cell pool and committed progenitors, and improves their in vitro functionality and in vivo engraftment. This observation may complement the existing protocols used in the manipulation of hematopoietic cells for therapeutic purposes. These findings may have an impact in the CB transplant procedures involving a combined infusion of unmanipulated and expanded grafts.

## Introduction

Cord Blood serves as an alternate source of hematopoietic stem cells for patients with malignant and non malignant conditions for whom HLA matched donors are not available. Cord blood possesses several inherent advantages over the bone-marrow–derived hematopoietic stem cells like the ease of procurement and low risk of severe graft-versus-host disease (GVHD) [1]. Unfortunately despite these attractive features, cord blood transplantations for adult patients still lag behind due to a low number of nucleated cells and CD34^+^ cells within a single cord blood collection. Double cord transplantation and a combined infusion of an unmanipulated and an expanded graft have been tried in clinics to tackle this problem [2], [3], [4], [5]. Thus the strategies to expand either CD34^+^ cells or the selected subpopulations from cord blood are an area of active research. Last few years witnessed various clinical trials in the transplantation of expanded graft and have demonstrated the safety and the feasibility of the expansion procedures [6], [7], [8], [9]. However in majority of cases the common problem confronted was the altered behavior of the cultured hematopoietic stem/progenitor cells (HSPCs) making the expanded graft less competent in transplant settings. The expansion culture is known to cause several cellular defects like loss of stem cells, down regulation of adhesion/migration properties, reduced clonogenicity and initiation of apoptosis [8]. This may render the already fewer stem cells compromised causing an altered marrow engraftment. Some of the earlier studies have pointed out the role of apoptosis cascade in maintaining the stem cell compartment [10], [11]. The role of two cysteine proteases; caspase and calpain has been highly implicated in programmed cell death in many cell systems [12], [13].

The impact of apoptosis in the hematopoietic compartment has been pointed out by Liu and colleagues as they demonstrated an engraftment defect, when the cultured CD34^+^ cells were transplanted into the SCID model due to the activation of the apoptotic CD95 pathway [14]. Our previous studies also suggested a negative impact of apoptosis on the behavioral aspects of frozen mouse bone marrow cells [15]. The pan caspase inhibitor zVADfmk is used in cryopreservation studies as well [16]. More over, we have consistently observed a three to four fold increase in apoptosis upon cytokine stimulation of CB derived CD34^+^ cells compared to their fresh counterparts.

Keeping these findings in mind, we hypothesized that the prevention of apoptosis may play an important role during the ex vivo expansion of HSPCs. To validate this hypothesis, we employed a strategy of transient regulation of apoptosis by using the cell permeable inhibitors of two major apoptotic proteases; caspase and calpian as supplements to the cytokine containing expansion medium. We observed a significant improvement in the CD34^+^ cell expansion upon protease inhibition. The expanded graft also exhibited superior in vitro and in vivo functional properties.

Our data reveals a novel role of apoptotic protease inhibitors in stimulating hematopoiesis in vitro, thereby improving the quality of expanded grafts. The observations herein may contribute to the expansion protocols when used either singly or in conjunction with other methods.

## Results

### Presence of zVADfmk and zLLYfmk improves the cell yield and reduces apoptosis during ex vivo expansion

We observed a higher incidence of apoptosis, when the cord blood derived CD34^+^ cells were cultured with cytokines compared to their freshly isolated counterparts (Figure S1). Thus we sought to assess the role of cell permeable inhibitors of cysteine proteases; caspase (zVADfmk) and calpain (zLLYfmk) as supplements in the serum free culture of CD34^+^ cells. We noticed that after the ex vivo culture, the total cell yield increased up to 30–70 fold, relative to the input cell number in all the three sets in various cord blood units analyzed (data not shown). However, the presence of zVADfmk or zLLYfmk resulted in a significantly higher cell yield compared to the control on the 10^th^ day (Figure S2A, n = 6).

**Figure 1:**
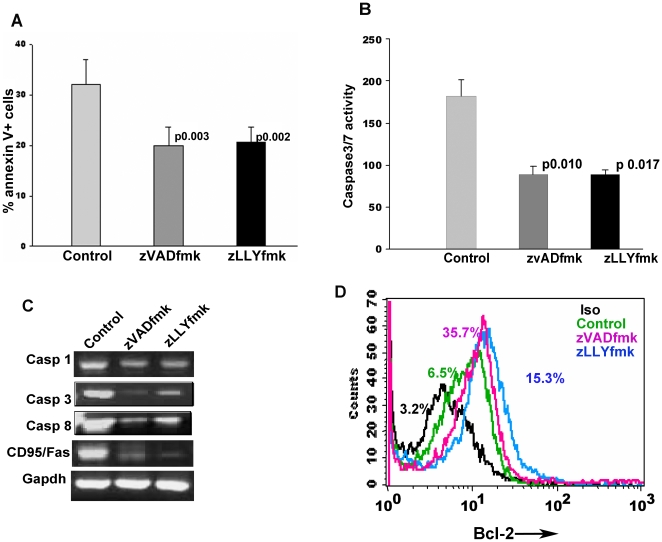
zVADfmk and zLLYfmk reduced the level of apoptosis during ex vivo expansion. (A) The expanded control and zVADfmk/zLLYfmk cells were assessed for the expression of pro and anti apoptotic molecules. Annexin V staining was carried out to detect the apoptotic population. The percent annexinV^+^ population decreased significantly when the CD34^+^ cells were cultured in the presence of either of the protease inhibitors showing a reduced level of apoptosis in the culture. Data represented as mean positive population ± standard deviation from five independent experiments n = 5 (B) The presence of active caspases (Casp3/7) in the culture was assessed using a cell free cytosolic enzymatic assay and the zVADfmk/zLLYfmk sets showed a significant reduction in the activity of these caspases as assessed from four different CB units. Data is represented as mean ± standard deviation. (n = 4). (C) The transcript analysis of various pro apoptotic genes like caspase 1, caspase 3, caspase 8 and CD95/Fas, revealed a reduction in their expression upon protease inhibition compared to the control. (D) The analysis for the presence of anti apoptotic bcl-2 showed a significant increase in the cells expanded with zVADfmk/zLLYfmk. Representative flowcytometry profile is shown. (Black line– Isotype, green line – control, pink line – zVADfmk and blue line- zLLYfmk respectively (n = 4).

Apoptosis in the culture was analyzed by a multiparametric study combining the protein and transcript analysis. Annexin V staining was carried out to detect the initial phase of apoptosis. A shown in Figure 1A, there was a significant reduction in the Annexin V+ population in the cells expanded with zVADfmk/zLLYfmk compared to the control (p0.003, p0.002, n = 5). We further observed that both the caspase and calpain inhibitors reduced the activation of caspases with an equal efficiency. The presence of active downstream/executioner caspases viz caspase3/7, was significantly reduced in the test sets as assessed from a cell free cytosolic enzymatic assay (Figure 1B, p 0.010, p 0.017, n = 4). Similarly, the transcript level analysis showed a reduction in the expression of major members of the apoptotic machinery. The expression of various genes for caspase1, caspase 3 and caspase 8 was studied along with Fas antigen/CD95 (Figure 1C). There was a 1.25 fold reduction in the ICE/casp1 mRNA in the test cells compared to control. The caspase 3 mRNA was down regulated 1.3–1.5 times, whereas caspase 8 was down regulated by 1.5 times in the test cells. The Fas/CD95 was down regulated 2 fold in the cells expanded in presence of zVADfmk, and was reduced 2.75 times in the cultures expanded in the presence of zLLYfmk.

Reduction in the apoptosis was accompanied by an increase in the bcl-2^+^ population. Figure 1D depicts the representative flowcytometry profile showing a higher bcl-2^+^ population. Since bcl-2 expression is known to augment the stem cell functionality, we compared the control and zVADfmk/zLLYfmk cultured cells that co express bcl-2 along with the CD34 marker. There was a substantial increase in the number of bcl-2^+^ cells within the CD34 compartment upon protease inhibition as revealed by a two colour flowcytometry analysis (Figure S2B, n = 4).

### Presence of cysteine protease inhibitors favoured the expansion of CD34^+^ cells

**Figure 2:**
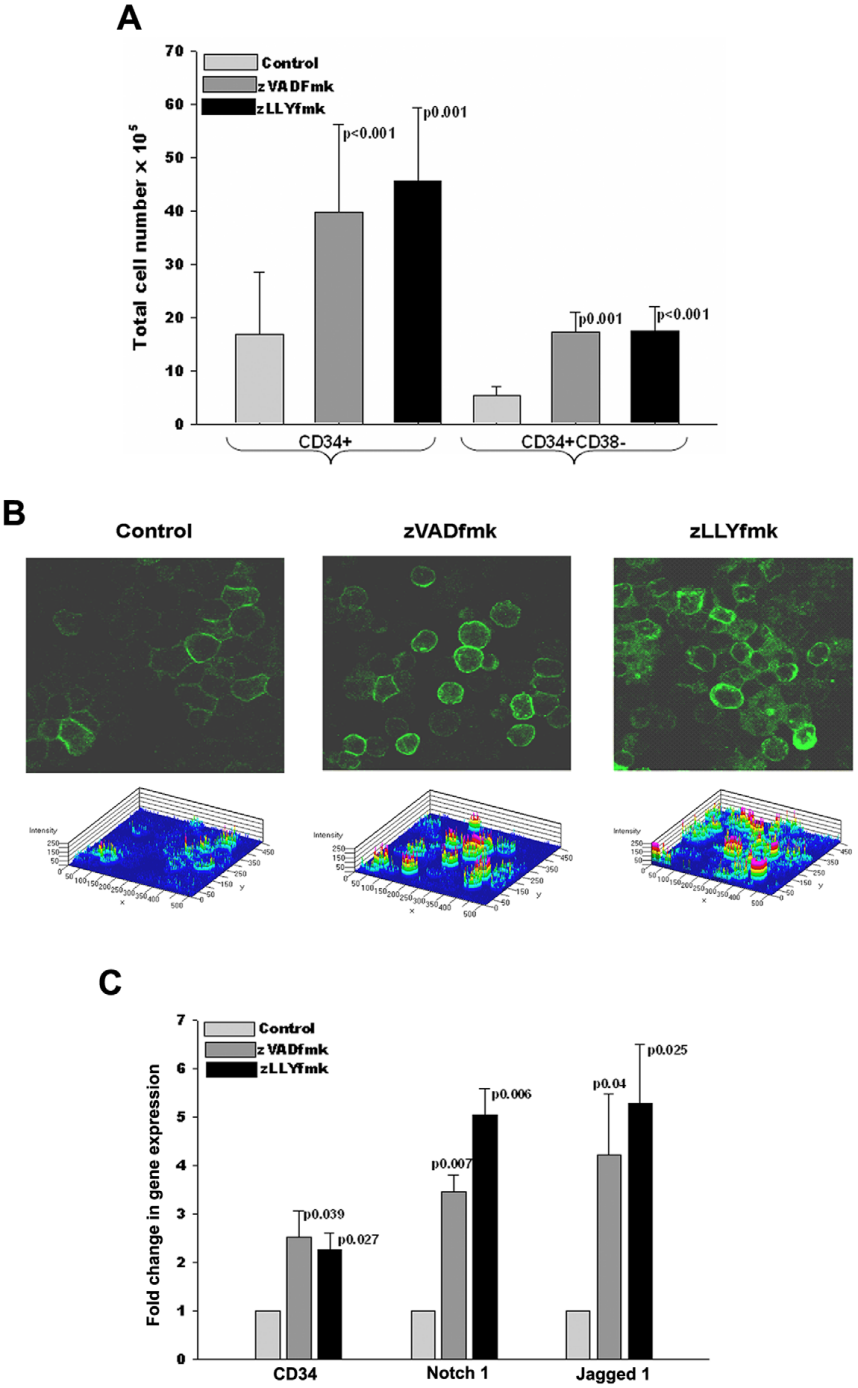
Presence of cysteine protease in the culture favored the expansion of CD34^+^ cells. (A) The expanded control and zVADfmk/zLLYfmk exposed HSPCs were stained using anti human CD34 antibody and was quantitated by flowcytometry. The percent positive population was normalized to the respective cell yield on the 10^th^ day (n = 5). The CD34^+^CD38^−^ cell content of the expanded test HSPCs was significantly increased. (B) Collected HSPCs were immunostained using anti human CD34 antibody and analyzed by confocal microscopy. There was a remarkable increase in the CD34 intensity when the cells were expanded in the presence of the protease inhibitors showing that the inclusion of these compounds increased the basal CD34 expression. Bar = 10 µm. (C) Quantitative real-time analysis showing a higher gene expression of CD34, notch1 and jagged 1 in the test expanded HSPCS relative to the control.

The expansion efficiency mainly relies on the CD34^+^ cell content of the graft. The addition of zVADfmk or zLLYfmk during the in vitro expansion significantly increased the total CD34^+^ cell content compared to the control (Figure 2A). More over there was a 3 fold increase in the CD34^+^CD38^−^ cell content in the presence of either the compounds (Figure 2A, n = 5).The surface CD34 expression at individual cell level was remarkably increased in the test sets as confirmed from confocal laser microscopy, implicating a role of these two compounds in the modulation of CD34 turn over (Figure 2B, n = 3).

To corroborate the above observation of enhanced generation of CD34^+^ cells in culture, we analyzed the relative abundance of the gene expression of CD34 in the both the experimental conditions. The quantitative real time PCR analysis revealed a higher transcript level of CD34 in the presence of zVADfmk/zLLYfmk compared to the control confirming the above observations. (Figure 2C, n = 3). These findings were noteworthy as it unveiled a hitherto unknown role of two known apoptotic inhibitors in regulating the CD34^+^ population at both the protein and transcript levels.

Apart from increasing the CD34 gene expression, the presence of zVADfmk and zLLYfmk up regulated the notch receptor 1(3–5 fold), and its ligand jagged 1 (4–5 fold) which is one of the crucial signaling pathway involved in the maintenance of stem cell compartment in the hematopoietic system. These results indicated that despite the conventional role of reducing the apoptosis level, these compounds extended their function in positively regulating the stem cell pool in the expanded cells by activating the notch signaling. (Figure 2C, n = 3).

### The expanded HSPCs were predominantly myeloid committed in nature

**Figure 3:**
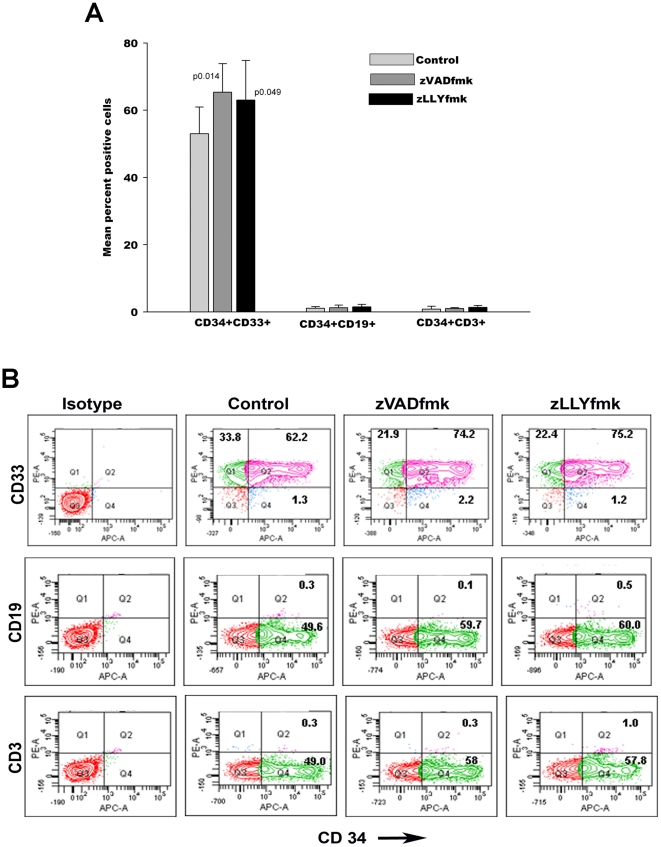
Lineage assessment of the expanded HSPCs. (A)The expanded HSPCs were double immunostained with CD34 vs. lineage antibodies like CD33 (myeloid) CD19 (B lymphoid) and CD3 (T lymphoid) cells. 50–70% of cells comprised of CD34^+^CD33^+^ myeloid with a negligible percentage of CD34+CD19^+^ and CD34^+^CD3^+^ cells. The zVADfmk/zLLYfmk expanded cells showed a significant increase in the CD34^+^CD33+ subset as compared to the control. (B) Representative flowcytometry profile showing the double Immunostaining of CD34 vs CD33, CD19 and CD3.

In order to check whether the incorporation of the protease inhibitors leads to a proliferation associated differentiation, we analyzed the expanded cells for the presence of lymphoid and myeloid lineages. We observed that 50–70% the expanded CD34^+^ cells co-expressed the myeloid marker CD33 and a remarkable increase in the myeloid progenitor population (CD34^+^CD33^+^) was observed in the zVADfmk/zLLYfmk cultured cells compared to the control indicating the presence of more progenitor cells upon protease inhibition (Figure 3A, p0.014, p0.049, n = 3). Moreover, the negligible percentage of CD34^+^CD33^−^ subset ruled out the possibility of the binding of mature myeloid cells to CD34. This was assessed by the double immunostaining of CD34 vs.CD33.The data further revealed that virtually all the cells examined were CD34^+^19^−^ (B lymphoid) and CD34^+^CD3^−^ (T lymphoid) after the culture period. Figure 3B depicts a representative flowcytometry profile of the same.

### Enhanced colony formation of the cells expanded in the presence of protease inhibitors

**Figure 4:**
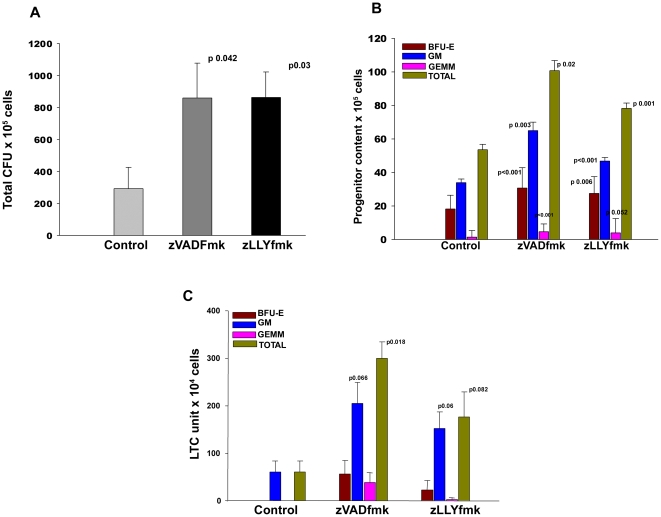
Improved CFU and LTC output of the expanded HSPCs. The in vitro functional properties of the expanded HSPCs significantly improved upon protease inhibition. (A) Colony formation assay on a semi- solid methyl cellulose media was carried out. After 14 days of incubation, the three major colonies (BFU- E, GM, and GEMM) were scored under an inverted microscope. The total colonies were normalized to the cell yield. There was a 3.5 fold increase in total progenitors in the cells expanded in the presence of zVADfmk and zLLYfmk. Data are represented as mean ± standard deviation of four different CB samples (n = 4). (B) A representative data showing the presence of differential colonies: BFU E, GM and GEMM formed in the three sets. (C) LTC assay was carried out to assess the presence of more primitive stem cells within the cultured HSPCs. At the end of fifth week, the adherent and non adherent cells were pooled and subjected to colony formation and the major colonies formed were scored. The cells expanded in the presence of either of the protease inhibitors preserved the LTC units to a higher extent. There were no BFU-E and GEMM colonies in the control. The zVADfmk/zLLYfmk sets formed both BFU-E and GEMM in addition to GM colonies. Data from a representative experiment is shown (n = 3).

In vitro colony formation reveals the presence of early progenitors in the culture, which can differentiate into myeloid or lymphoid colonies in the presence of a combination of growth factors. The CD34^+^ cells expanded with and without apoptotic inhibitors were seeded in semi solid methyl cellulose medium with the growth factor combinations as described in methods. The multipotential differentiation ability of HSPCs in the methyl cellulose assay showed a significant increase in the total and differential colony formation in the presence of either of the compounds compared to the control. Figure 4A is the mean data of 4 different samples showing a 3.5 fold increase in the total CFU units in the zVADfmk/zLLYfmk sets. (p0.042, p 0.03, n = 4). Differential colony count of a representative sample is shown in Figure 4B showing an increase in all the three major colonies (Burst forming unit erythroid- BFU E, Granulocyte-macrophage-megakaryocytes – GEMM and granulocyte-macrophage–GM) in the presence of inhibitors.

### The cells expanded in the presence of protease inhibitors contained higher number of Long term culture initiating cells

The LTC system not only offers an approach to investigate the proliferative and differentiative events, but also reflects the self-renewal ability of HSCs. The expanded control and zVADfmk/zLLYfmk exposed HSPCs were cultured on an irradiated supportive stromal feeder layer for further five weeks followed by colony forming assay. The cells expanded in the presence of the compounds retained significantly higher number of primitive population (LTC units) compared to the control sets. The data of a representative sample is shown in Figure 4C.There was a 2.5–3.5 fold increase in the number of GM colonies in the cells expanded in presence of inhibitors. Moreover, these cells generated the erythroid and CFU mix colonies whereas the control cells formed only the GM colonies. The presence of GEMM colonies, in comparison to the control, signifies the preservation of more primitive cells in those sets. Similar increase in total LTC units was observed with different expanded cord blood units (Figure S2C, n = 3).

### Protease inhibition improved the engraftment of ex vivo expanded HSPCs

**Figure 5:**
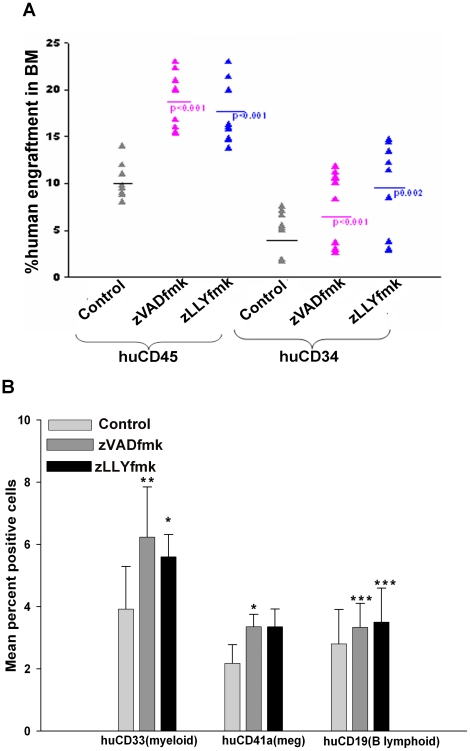
In vivo engraftment of the expanded HSPCs in NOD/SCID mice. (A) The SRC (Scid Repopulating Cells) efficiency of the cultured HSPCs was assessed in NOD/SCID mouse model. The expanded cells were intravenously administered to the sub lethally irradiated recipient. After 16th week (4 months), the animals were sacrificed and the bone marrow cells were stained for human pan leukocyte marker CD45 and progenitor marker CD34. The presence of zVADfmk/zLLYfmk in the expansion culture proved to be more efficient in repopulating the marrow compared to the control as there was a significantly higher engraftment detected by huCD45 and huCD34 (B) Multilineage engraftment potential of the zVADfmk/zLLYfmk expanded cells was superior to their control counterparts as detected from the presence of huCD33, huCD41a and huCD19 in the bone marrow of recipient mice. Data represented as mean ± standard deviation (n = 3).

To assess the in vivo engraftment ability of the expanded graft, we intravenously administered the cultured control and test cells into sub lethally irradiated NOD/SCID mice as described in methods. 10^6^ cells of the expanded end product were used for intra venous infusion. The animals were sacrificed on 16^th^ week post transplant and the level of human engraftment was assessed. The percent engraftment in each set was compared to the PBS infused irradiated recipient to detect the background staining if any. Both the expanded control and zVADfmk/zLLYfmk expanded cells engrafted the irradiated recipient. However, the animals, which received the HSPCs, expanded in the presence of either of the inhibitors showed a higher engraftment than the animals that received the HSPCs expanded with growth factors alone. These superior engraftment potential correlated well with the in vitro results. Figure 5A shows the engraftment of huCD45 and huCD34 in the bone marrow of individual animals revealing the superior potential of the zVADfmk/zLLYfmk expanded graft compared to the standard cytokine expanded graft (p≤0.001, p0.001).The representative flow contour plots are shown in Figure S3A and Figure S3B.The multilineage engraftment was assessed by using a panel of antibodies against lympho-myeloid lineages. The presence of zVADfmk or zLLYfmk was found to be beneficial in generating a higher myeloid (CD33), megakaryocytic (CD41a) and lymphoid (CD19) population in the bone marrow of the irradiated recipient (Figure 5B). The representative flow contour plots are shown in Figure S3C–E.

### The presence of protease inhibitors in the media was also beneficial for the expansion of frozen-thawed cord blood units

**Figure 6:**
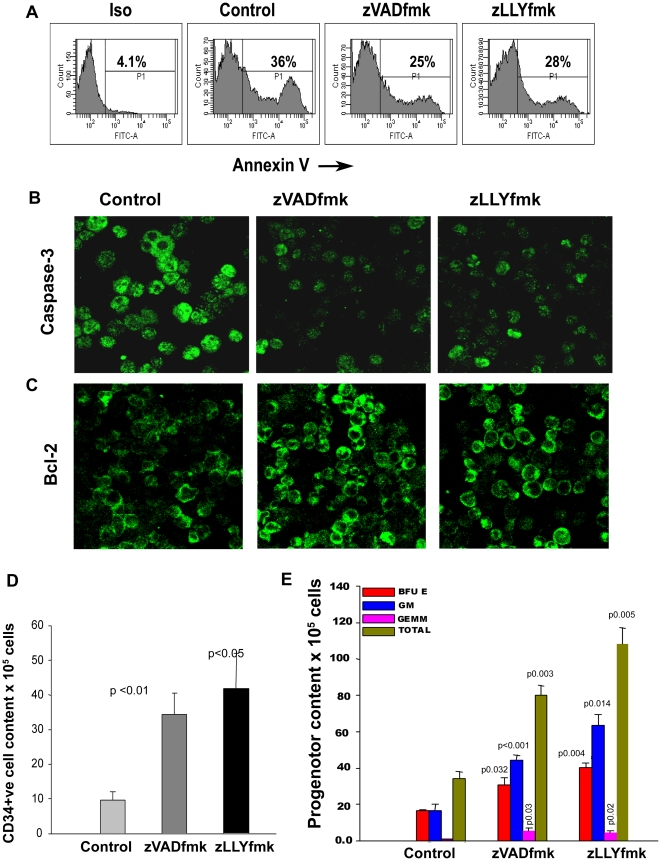
The presence of protease inhibitors in the media was also beneficial for the expansion of frozen-thawed CB units. (A) Representative flowcytometry profile showing a reduced number of Annexin V^+^ population when the frozen CD34^+^ cells were expanded in the presence of either of the compounds, n = 3. (B) The cytosolic caspase 3 expression was remarkably reduced in the zVADfmk/zLLYfmk sets compared to the control counterpart as analysed by confocal microscopy. (C) Concomitantly the bcl-2 expression in those cultures increased relative to the control, showing a reduction in the apoptosis cascade. Results presented are representative fields confirmed from at least 3 different samples. Bar = 10 µm. (D) The percent CD34^+^ population was quantified by FACS and it was observed that CD34^+^ cell content increased (3.5–4 fold) fold when the frozen thawed CB units were expanded in the presence of either of the compounds (n = 4). (E) The clonogenic potential of the test expanded cells showed a significant increase in the differential colony formation. The presence of GEMM colonies were remarkably higher compared to the control frozen cells. Data of a representative experiment is shown (n = 4).

Cord blood transplantations in the clinics mainly involve the use of cryopreserved grafts. Our current observations with fresh cord blood units clearly suggested that an optimized anti apoptotic strategy in frozen units will have a significant and positive impact during expansion, thereby potentially improving their clinical utility. In order to test this hypothesis, we carried out the expansion of frozen CD34^+^ cells with and without the two compounds and found that the two protease inhibitors were equally effective. Apoptosis was found to be reduced in the zVADfmk/zLLYfmk cultured cells. AnnexinV^+^ population and caspase-3 expression was reduced as shown in Figure 6A and Figure 6B (n = 3). Concomitantly a higher bcl-2 expression was seen in those cells compared to the control (Figure 6C, n = 3). There was an increase in the CD34^+^ cell content up to 3.5–4 fold in the presence of zVADfmk/zLLYfmk relative to the control (Figure 6D, n = 3). The clonogenic potential was also assessed and found to be significantly higher as shown in Figure 6E (n = 4).

These observations prompted us to assess the possibility of cryopreservation of the expanded HSPCs from fresh CD34^+^ cells. Post thaw analysis revealed a higher recovery of both viable and nucleated cells from the cells expanded in the presence of these compounds. The CD34^+^ population was maintained to a significantly higher extent along with the primitive CD34^+^CD38^−^ subset. This reflected in the higher colony formation and LTC output (data not shown), thus underscoring the fact that the beneficial effect of these two additives during expansion, persisted even after the freezing stress.

Overall, our findings highlight the potential use of these two compounds in the expansion protocols so as to yield a superior quality graft which may benefit in transplant settings.

## Discussion

Cord blood is an established source for stem cell transplantation procedures. In contrast to marrow, CD34^+^ cells from cord blood possess higher proliferative potential, and a higher frequency of long term and SCID repopulating cells [17]. However, the limited number of cell dose in this source has prompted researchers to develop alternative methods to increase the cell number. Ex vivo expansion is one approach to increase the number in a clinically relevant manner [18]. This strategy overcomes the limitation posed by low cellularity of CB units for unrelated transplants in adults. Development of reliable methods for the expansion of cord blood stem cells is critical to ensure their further clinical application. Many investigators have employed various methods to expand the HSCs; either by genetic/epigenetic approaches or by using specific compounds which affect self renewal pathways [19], [20], [21], [22]. Tanaka H et al. has used a Hox decoy peptide for expansion and reported a nearly 2 fold increase in the CD34^+^ cells [20]. Use of valproic acid has also resulted in a 2 fold expansion of CD34^+^ cells as shown by Seet et al [22]. Recently Nishino and colleagues have used a c- MPL agonist to expand the CD34 cells [21]. Several groups have reported varying cell yield depending on the source of isolated cells and the culture conditions [23], [24] Ueda and colleagues have amplified HSPCs in serum free medium and found that the combination of SCF, TPO, Flt3-L IL-6 and IL-6-R as growth factors was superior in expanding SRCs. They demonstrate a 45 fold increase in total cell number [25]. We report a 30–70 fold increase in total nucleated cell yield in all three sets after ex vivo expansion using a similar combination of cytokines used by Ueda et al except for IL-6R. However, the concentrations used were much lower than those used by them. The use of Stem pro media which is formulated to support the amplification of HSCs might have also contributed for the robust cell expansion even in control cells. [26] The experimental ‘control’ in our cultures, thus served as a normal expanded graft. Keeping this ‘control’ as background we have further aimed to increase the quality and functionality of the expanded HSPCs by supplementation of apoptotic protease inhibitors.

Here we demonstrate for the first time that the reduction in the level of apoptosis brought about by zVADfmk and zLLYfmk during the ex vivo culture of HSPCs, increases the CD34^+^ cell content and enhances the functional properties of the graft. The maintenance of HSCs requires the coordination of complex pathways that involve control of self-renewal, differentiation and apoptosis as reviewed by Alenzi et al [27]. Of note, Hatzold and colleagues have revealed a striking functional link between apoptosis and asymmetric cell division in C.elegans [28]. Their studies point out to an evolutionary conserved role of apoptosis in stem cell biology. Furthermore another body of research highlighted that the over expression of anti apoptotic bcl-2 maintained the mouse embryonic stem cells in an undifferentiated state in a feeder free condition [29]. Domen and colleagues have shown that a block in apoptosis has increased the HSC pool in bcl-2 transgenic mice [10]. An elegant work by Imai et al in murine system showed that the use of zVADfmk facilitated the engraftment of mouse marrow cells in an intra bone marrow transplantation [30]. These evidences link the negative regulation of apoptosis with the maintenance of stem cell pool.

Our observations with cord blood derived CD34^+^ cells also indicate that a similar regulatory role of apoptosis exists in the human progenitor compartment as well. The supplementation of the expansion media with the protease inhibitors, did not cause an enhanced differentiation, but preferentially expanded the CD34^+^ population, overcoming the major hurdle in the expansion protocols. CD34 expression assessed by confocal microscopy revealed that both surface and cytoplasmic pattern (data not shown) of CD34 existed. However the intensity was consistently higher in test sets as described in results. Higher CD34^+^ cell content is important in the light of transplantation settings. Moreover we observed a three fold increase in CD34^+^CD38^−^ cell content during expansion in the presence of either of the compounds. Since the regulation of apoptosis cascade was a transient phase in our studies, the DNA damaged cells are expected to die off after the withdrawal of inhibitors. Thus the survival of abnormal stem cells which may be a concern from the translation point of view may be minimal in our cultures. However further studies are required to address this possibility.

Earlier studies have shown the significance of notch, and its ligands, in the expansion and self renewal of HSPCs [31], [32]. The recent preclinical and clinical observations by Delaney et al revealed that notch mediated expansion results in marked increase in the stem/progenitor cells and the SRCs [33]. Our observation of the up regulation of gene expression of the notch receptor and its ligand jagged 1 suggested that the addition of these two compounds during expansion, activated the notch signalling molecules. In this context, it would be interesting to explore the other signalling pathways also which has a direct role in the regulation of the stem cell pool.

The increased bcl-2 expression evident in zVADfmk/zLLYfmk sets is functionally relevant as bcl-2 has been reported to augment the stem cell functionality [34]. Janzen et al have shown that caspase-3 null mice harbour long term hematopoietic stem cells to a higher extent compared to the wild type animal [35]. The negative regulation of caspases has also been shown to be necessary for the pluripotent state of embryonic stem cells [36]. All these observations speculated a possible role of caspases and other cysteine proteases in the maintenance of stem cell turn over. We demonstrate that the increased expansion efficiency of cord blood CD34^+^ cells in the presence of anti apoptotic agents was associated with a concomitant reduction in the activity of major caspases. The down regulation of Fas antigen is relevant here as it is shown to inversely correlate with the homing efficiency of CD34^+^ cells [14]. More over, Bryder D et al have demonstrated a negative impact of Fas mediated apoptosis in the self renewal of hematopoietic stem cells [11]. However, whether there is a possibility of faster senescence/shorter life time due to a higher proliferation rate (upon apoptosis inhibition) needs to be further looked into.

We further show the beneficial effect of protease inhibition on the functional properties of the expanded cells. The multipotent colony forming ability of the test cells was remarkably higher compared to the control sets as assessed by colony formation and LTC assays. The immature population of GEMM colonies increased significantly in both the assays indicating an increased survival and preservation of stem cells in the expanded test sets. Our experiments with frozen thawed cord blood units demonstrated the equal effectiveness of these compounds during the in vitro expansion underscoring their clinical utility.

Expansion has been reported to compromise on the engraftment potential of HSCs. Expansion of both HSCs and HPCs are essential, since the committed progenitors give rise to an immediate short term engraftment where as the primitive HSCs in the graft sustain hematopoiesis throughout [37]. The myeloid restricted HSPCs are critical for the initial phase of clinical transplantation as observed by several investigators [17], [38]. The end product of our ex vivo cultures were predominantly myeloid committed in nature with a significantly higher CD34^+^ cell content in the presence of zVADfmk/zLLYfmk which resulted in an improved engraftment in animals that received those grafts (>0.1% positive human cells were considered as a cut off for engraftment as per Mattia et al [39] ).The presence of lymphoid (huCD19) engraftment in the bone marrow using cord blood expanded HSPCs are reported by a few investigators [25], [40]. In our studies, the engraftment in NOD/SCID mice was carried out for a period of 16 weeks, which is a permissive duration for the lymphoid engraftment to be seen, albeit less prominent compared to the engraftment detected by huCD45 and huCD34 markers. The higher number of immature GEMM (CFU mix) colony formation ex vivo upon protease inhibition, very well translated in regenerating a higher multilineage engraftment in NOD/SCID mice.

Taken together our results unveil a novel role of two apoptotic inhibitors; zVADfmk and zLLYfmk in stimulating hematopoiesis in vitro and improving the engraftment potential in vivo, making the expanded graft superior to the cytokine cultured HSPCs. Our findings may help in adding a further step in refining the expansion protocols to yield a graft competent in transplant settings.

## Methods

### Collection of Umbilical cord blood and isolation of CD34^+^ cells

Cord blood samples were collected from local hospitals with written informed consent from the mother. The institutional review board (IEC-Institutional ethical committee, NCCS) approved protocols for the uses of human samples were followed. Mononuclear cells were separated from the cord blood by ficoll hypaque (density 1.077 g/ml, Sigma Aldrich, St.Louis MO) density gradient centrifugation. CD34^+^ cells were isolated from the mononuclear cells using Dynal beads as per manufacturer's instructions (Dynabeads M-450 CD34; Dynal, ASA, Oslo, Norway).

### Ex vivo expansion of CD34^+^ cells in serum free medium

CD34^+^cells were seeded at a density of 5×10^4^ cells/well/500 µl of Stempro medium (GIBCO, Grand Island, NY), in 24 well plates. Cytokines, IL-6, SCF, TPO, and Flt-3-L (all growth factors were from Peprotech Inc, Rocky Hill, NJ) were used at a final concentration of 25 ng/ml with (test cells) and without (control cells) the addition of either zVADfmk -100 nM or the zLLYfmk 10 µM (MP Biomedicals, Aurora, Ohio). Cells were cultured for 10 days and were collected from the suspension cultures and centrifuged (1000 rpm for 5minutes). Further the cells were quantitated for expansion and apoptosis by various methods. After expansion, these cells were either used for assessing the invitro functionality by CFU, and LTC assay or were used for in vivo engraftment in NOD/SCID mice. Thus in vitro and in vivo studies were carried out using independent CB units.

### Flowcytometry analysis

Phenotypic analyses of the expanded cells were carried out at the 10^th^ day. Cells were analyzed for the expression of surface markers like Annexin V, CD34, CD38, Bcl-2, CD33, CD19 and CD3 (BD pharmingen- San Jose, California). The expanded control and treated cells were collected and suspended in 1xPBS containing 0.1% BSA. After the addition of antibodies the cells were incubated for 40 min on ice. Excess antibody was removed by washing with PBS and the cells were fixed using 1% paraformaldehyde solution and kept at 4°C till acquiring. Intracellular bcl-2 expression was analysed using hamster anti human antibcl-2 antibody (Beckton Dickinson; San Jose, California). For this the cells were fixed and permeabilized using BD fixperm kit (BD pharmingen -San Jose, California) and the staining was done as per manufacturer's instructions. Appropriate isotype controls were kept for each set. For Annexin V staining, the washed cells were suspended in 1× Binding buffer and 5 µL of Annexin V FITC, (BD pharmingen -San Jose, California) for 15 minutes at room temperature. 10 µl of PI solution was then added and analysed by flowcytometry. Total cells in the FSC and SSC pattern were gated after excluding the debris. A minimum of 10,000 events were for each sample on either FACS CantoII (Beckon Dickinson San Jose California) using FACS Diva software or on FACS Calibur (Beckon Dickinson San Jose California) using cell quest pro software.

### Immunofluorescence and Confocal microscopy

Ex vivo expanded HSPCs were analyzed for the expression of CD34 and the apoptosis related proteins like caspase 3 and bcl-2. Mouse antihuman CD34 antibody (BD pharmingen, San Jose, California), mouse antihuman bcl-2 and rabbit anti human caspase-3 (Santacruz biotech, Santacruz, CA, USA) antibodies were used at 1∶50 dilutions. Goat anti rabbit polyclonal IgG FITC and goat anti mouse monoclonal IgG FITC (BD pharmingen, San Jose, California) were used at 1∶100 dilution. Cells were fixed in 4% paraformaldehyde, permeabilized with chilled 50% methanol (as per requirement) and blocked with PBS containing 1% BSA for 1 hour. Primary antibodies were incubated overnight at 4°C, washed with PBS and then incubated with the secondary antibodies at room temperature for 1 hour. Slides were washed in PBS twice and mounted in mounting medium. Confocal images were obtained using a Zeiss LSM 510 laser scanning microscope. Results presented are representative fields confirmed from a minimum of 3 different biological samples.

### Cellular Caspase 3/7 activity assay

After 10 days of culture, the control and the zVADfmk/zLLYfmk exposed cells were collected and were subjected to lysis to obtain the cell free cytosolic fraction, as per the assay kit instructions. The Z-DEVD-R110 substrate based assay was carried out as per the manufacturer's instructions (Molecular Probes, Eugene, Oregon, USA).

### Reverse Transcription Polymerase chain reaction

mRNA was isolated from the expanded cells using Dynabeads mRNA DIRECT kit as per the manufacturers instructions (Invitrogen, Dynal ASA, oslo Norway). The eluted mRNA were reverse transcribed to cDNA by using reverse transcriptase (Sigma Aldrich, St Loius, MO) and oligo-dT primers (invitrogen) as per the instructions and PCR was performed with specific primers. The thermal cycle used was (95°C for 2 min, 95°C for 45 sec, annealing (as per different genes) for 45 sec, extension 72°C for 2 min) for 35 cycles and a final extension at 72°C for 5 minutes. The amplified product was run in 2% agarose gel and stained with EtBr (Ethidium bromide). The images were captured in a gel documentation system (Syngene Ingenius, NJ 08830, USA). The densitometric quantification of the amplified PCR products were done using Quantity One software (BioRad, Philadelphia, PA). The primer sequences used were as described in Figure S4.

### RNA isolation and Real-time PCR analysis

Total RNA was isolated using Trizol reagent (Sigma Aldrich, St Loius MO) as per manufacturer's instructions. The isolated RNA was quantitated using nanodrop spectrophotometer (ND1000). First strand cDNA synthesis was carried out by using ‘high capacity cDNA archive kit’ (Applied Biosystems, Foster City, CA) with random hexamers. 1 µg RNA of each sample was reverse transcribed to cDNA in 10 µl of final volume. 1 µl of the prepared cDNA was used for real-time PCR of each gene. The real time PCR was performed using the SYBR-Green PCR master mix (Applied Biosystems, Foster City, CA) and specific gene primers in a 7500 ABI-prism sequence detection system (Applied Biosystems).The final reaction volume for real time PCR was 20 µl for each gene and the thermal cycle used was 50°C for 2 min, 95°C for 10 min, (95°C for 15 sec, 60°C for 1 min) for 40 cycle. Samples without reverse transcription were used as negative controls. All qRT-PCR values were normalized to 18 s and calculated according to manufacturer's instructions.

### Colony Forming Unit assay

CFU assays were performed using the semisolid methyl cellulose media as described earlier [41]. After 14 days of culture, colony forming unit of Granulocytes-Macrophages (CFU-GM), Burst Forming Unit Erythroids (BFU-E), and mixed colonies of Granulocyte- Erythroid-Macrophage- Megakaryocytes (GEMM) were scored under an inverted microscope.

### Establishment of long term cultures

The LTC system allows the detection of very primitive HSPCs in the culture system. The LTC assay was carried out as described earlier [42]. After the 5 week culture period, the non-adherent and the adherent fractions were pooled and then were subjected to the colony formation assay.

### Engraftment of expanded cells in NOD-SCID mice

The NOD/LtSZ-scid/scid mice were obtained from Jackson Laboratories (The Jackson laboratory, 610 St.Bar Harbor, Maine 04609, USA) and were bred in the animal facility of our institute. The study was conducted adhering to the institution's guidelines for animal husbandry and has been approved by IAEC-NCCS/CPCSEA (Institutional animal ethical committee-NCCS/Committee for the Purpose of Control and Supervision of Experiments on Animals. Approval number: IACUC-Institutional Animal Care and Use Committee, EAF-Experimental Animal Facility/2004/B-71). Animal procedures involving intra-venous infusion were carried out under anesthesia causing minimal pain and distress to the animals. The approved protocol did not prohibit the use of anesthesia. During the experimentation period the animals were supervised under trained personals taking utmost care.

Mice at 4–6 weeks of age were exposed to sub lethal dose of 300 rads total body irradiation from a ^60^Co source (Gammachamber 5000, BRIT, Navi Mumbai, India). 10^6^ expanded cells from each set were mixed with an equal proportion of irradiated supportive cells taken from CD34 depleted fraction of non cultured cells, and were infused through the tail vein into the sub lethally irradiated mice (n = 3). Mice were sacrificed at 16^th^ week post transplant to assess the human engraftment.

The human engraftment in the bone marrow was detected by using huCD45 (pan hematopoietic) and huCD34 (stem/progenitor cells). The multilineage engraftment in the bone marrow was assessed by immunostaining the BM cells with a panel of antibodies against human lineage markers comprising CD33 (myeloid), CD41a (megakaryocyte), and CD19 (B lymphoid). All antibodies were purchased from BD pharmingen (Beckton Dickinson; San Jose, California).The cells were blocked with mouse IgG (Fc blocker) for 10 min at 4°C to reduce the non specific binding and were then incubated with the antibodies along with their respective isotype controls for 45 min at 4°C. In each experiment, one group of animals were kept which were irradiated and infused with PBS to detect the non specific background staining. RBCs were lysed with Lysis Buffer (Beckton Dickinson, San Jose, California) and the cells were washed twice with 1× PBS containing 0.1% BSA. The cells were then fixed using 1% paraformaldehyde in PBS and a minimum of 50,000 events were acquired on FACS CantoII (Beckton Dickinson, San Jose, California).

### Statistical analysis

The statistical differences between groups were analysed by one way repeated measure analysis of variance using the software SIGMA STAT (Jandel Scientific Corporation, San Rafael, CA) for all the experiments. All comparisons were between control vs zVADfmk and control vs zLLYfmk. The values were plotted as mean ± standard deviation Probability value: *p≤0.05, **p≤0.01, & ***p≤0.001 were considered statistically significant.

## Supporting Information

Figure S1 Apoptosis analysis of unexpanded and expanded HSPCs. Apoptosis was compared in the freshly isolated cord blood derived CD34+ cells to their cytokine cultured counterpart. The AnnexinV+ cells (apoptotic) increased 3–4 folds in the cultured counterpart implicating a higher incidence of apoptosis when the cells were cultured with growth factors alone in a serum free condition. An identical FSC/SSC gating strategy was followed for all samples on both day 0 and day 10.
(0.98 MB TIF)

Figure S2 Higher cell yield, bcl-2 expression and LTC output upon protease inhibition. (A) The total nucleated cell yield after the exvivo expansion. The presence of zVADfmk and zLLYfmk showed a higher cell yield compared to the control. Data are represented as mean ± standard deviation (n = 6) (B) Two colour flowcytometry analysis revealed the presence of a higher number of bcl-2+ cells in the CD34 compartment of the total HSPCs. The expanded HSPCs were immunostained for CD34. The stained cells were fixed and permeabilized and counter stained with anti bcl-2 antibody and analysed on FACS. The profile shows the percent bcl-2+ cells in the gated CD34 population. (C) Table summarizes the data of total LTC units formed from cultured control and test HSPCs, assessed from three different cord blood units. The total LTC output was significantly higher when the CD34+ cells were expanded in the presence of either of the anti apoptotic compound, revealing the presence of primitive stem cells in them. Data is represented as mean ± standard deviation (n = 3).
(2.79 MB TIF)

Figure S3 Human multilineage engraftment in the bone marrow of NOD/SCID mice. (A–B) Representative flowcontour plots showing the presence of higher huCD45 and huCD34 engraftment in the marrow of mice that received the cells cultured in the presence of zVADfmk/zLLYfmk (C–E) The human myeloid (CD33), megakaryocyte (CD41a) and B lymphoid (CD19) engraftment in the bone marrow.
(1.30 MB TIF)

Figure S4 Primer sequences used for PCR analysis. Table summarizes the list of the primer sequences used to perform the PCR reaction.
(0.64 MB TIF)
