# Correction: Expansion of Cord Blood CD34^+^ Cells in Presence of zVADfmk and zLLYfmk Improved Their In Vitro Functionality and In Vivo Engraftment in NOD/SCID Mouse

**DOI:** 10.1371/journal.pone.0092493

**Published:** 2014-03-10

**Authors:** The PLOS ONE Staff

The name of the first author is incorrectly given in the author byline. The correct name is: Sangeetha V.M. In addition, the first author’s name is incorrectly represented in the Citation. The correct Citation is: V.M. S, Kale VP, Limaye LS (2010) Expansion of Cord Blood CD34^+^ Cells in Presence of zVADfmk and zLLYfmk Improved Their In Vitro Functionality and In Vivo Engraftment in NOD/SCID Mouse. PLoS ONE 5(8): e12221. doi:10.1371/journal.pone.0012221
