# Supplementary figures and images for: Expansion of Cord Blood CD34+ Cells in Presence of zVADfmk and zLLYfmk Improved Their In Vitro Functionality and In Vivo Engraftment in NOD/SCID Mouse
Source: PLoS One. 2010 Aug 17;5(8):e12221. doi: 10.1371/journal.pone.0012221 (PMC2923186; doi:10.1371/journal.pone.0012221)

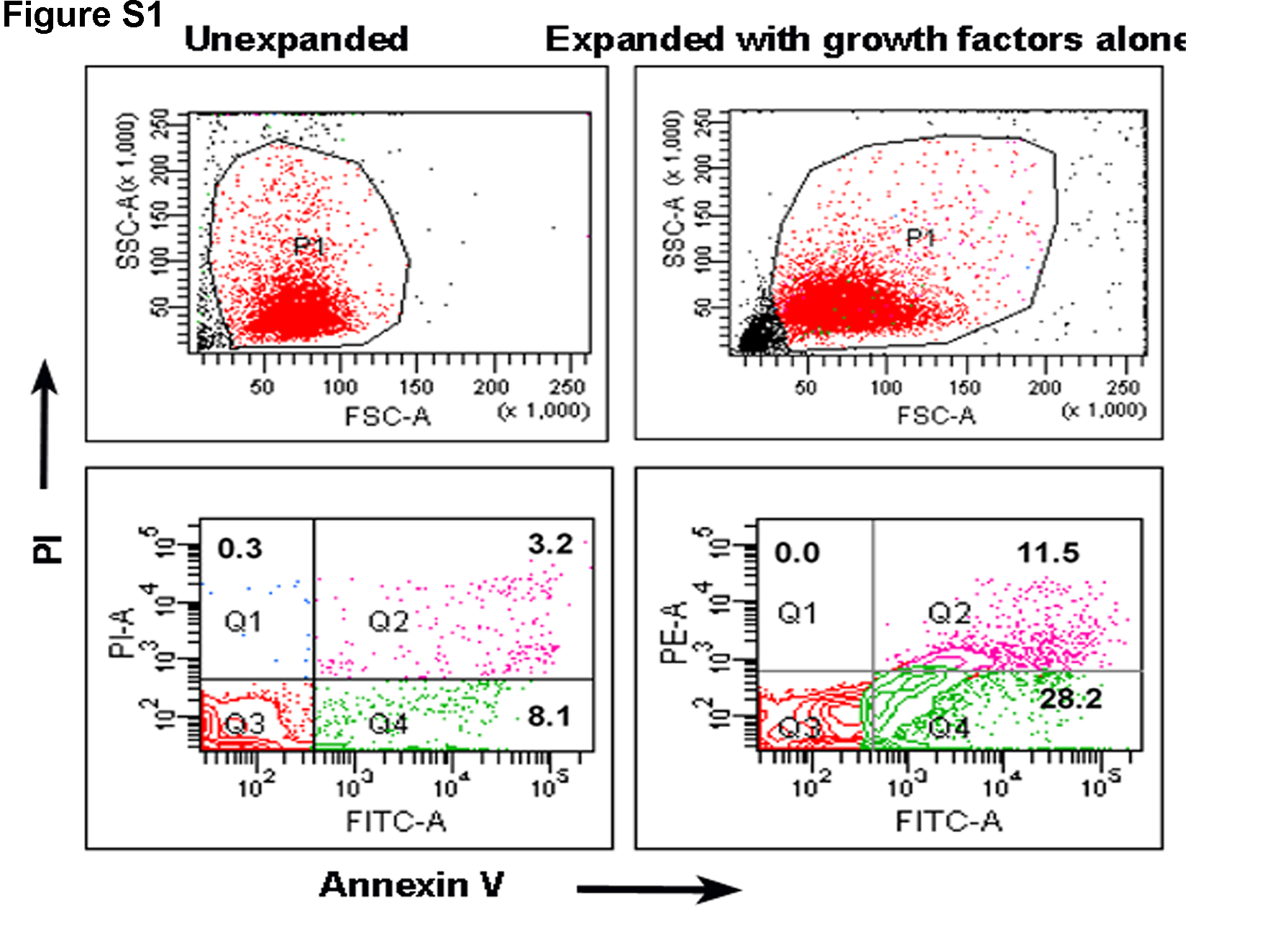

Supplement: Figure S1 — Apoptosis analysis of unexpanded and expanded HSPCs. Apoptosis was compared in the freshly isolated cord blood derived CD34+ cells to their cytokine cultured counterpart. The AnnexinV+ cells (apoptotic) increased 3–4 folds in the cultured counterpart implicating a higher incidence of apoptosis when the cells were cultured with growth factors alone in a serum free condition. An identical FSC/SSC gating strategy was followed for all samples on both day 0 and day 10. (0.98 MB TIF) [file pone.0012221.s001.tif]

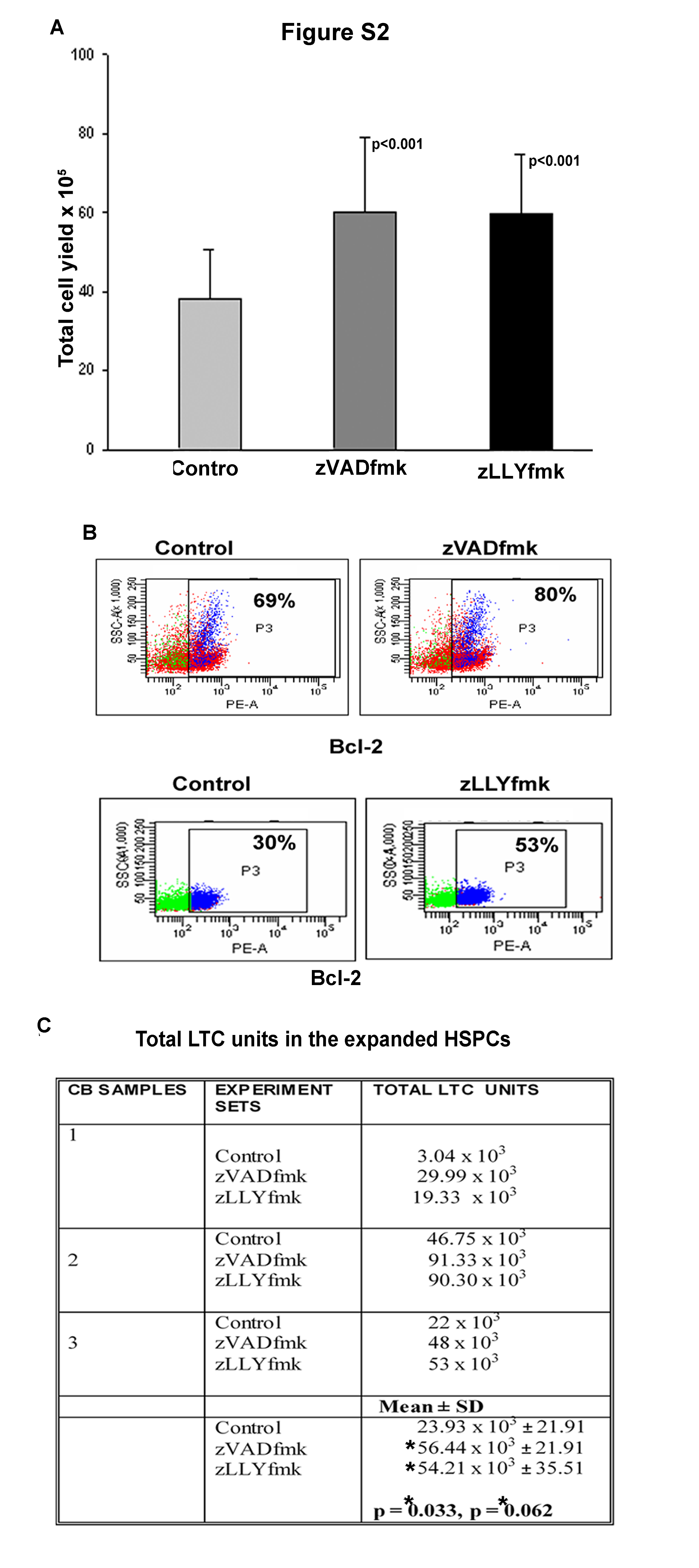

Supplement: Figure S2 — Higher cell yield, bcl-2 expression and LTC output upon protease inhibition. (A) The total nucleated cell yield after the exvivo expansion. The presence of zVADfmk and zLLYfmk showed a higher cell yield compared to the control. Data are represented as mean ± standard deviation (n = 6) (B) Two colour flowcytometry analysis revealed the presence of a higher number of bcl-2+ cells in the CD34 compartment of the total HSPCs. The expanded HSPCs were immunostained for CD34. The stained cells were fixed and permeabilized and counter stained with anti bcl-2 antibody and analysed on FACS. The profile shows the percent bcl-2+ cells in the gated CD34 population. (C) Table summarizes the data of total LTC units formed from cultured control and test HSPCs, assessed from three different cord blood units. The total LTC output was significantly higher when the CD34+ cells were expanded in the presence of either of the anti apoptotic compound, revealing the presence of primitive stem cells in them. Data is represented as mean ± standard deviation (n = 3). (2.79 MB TIF) [file pone.0012221.s002.tif]

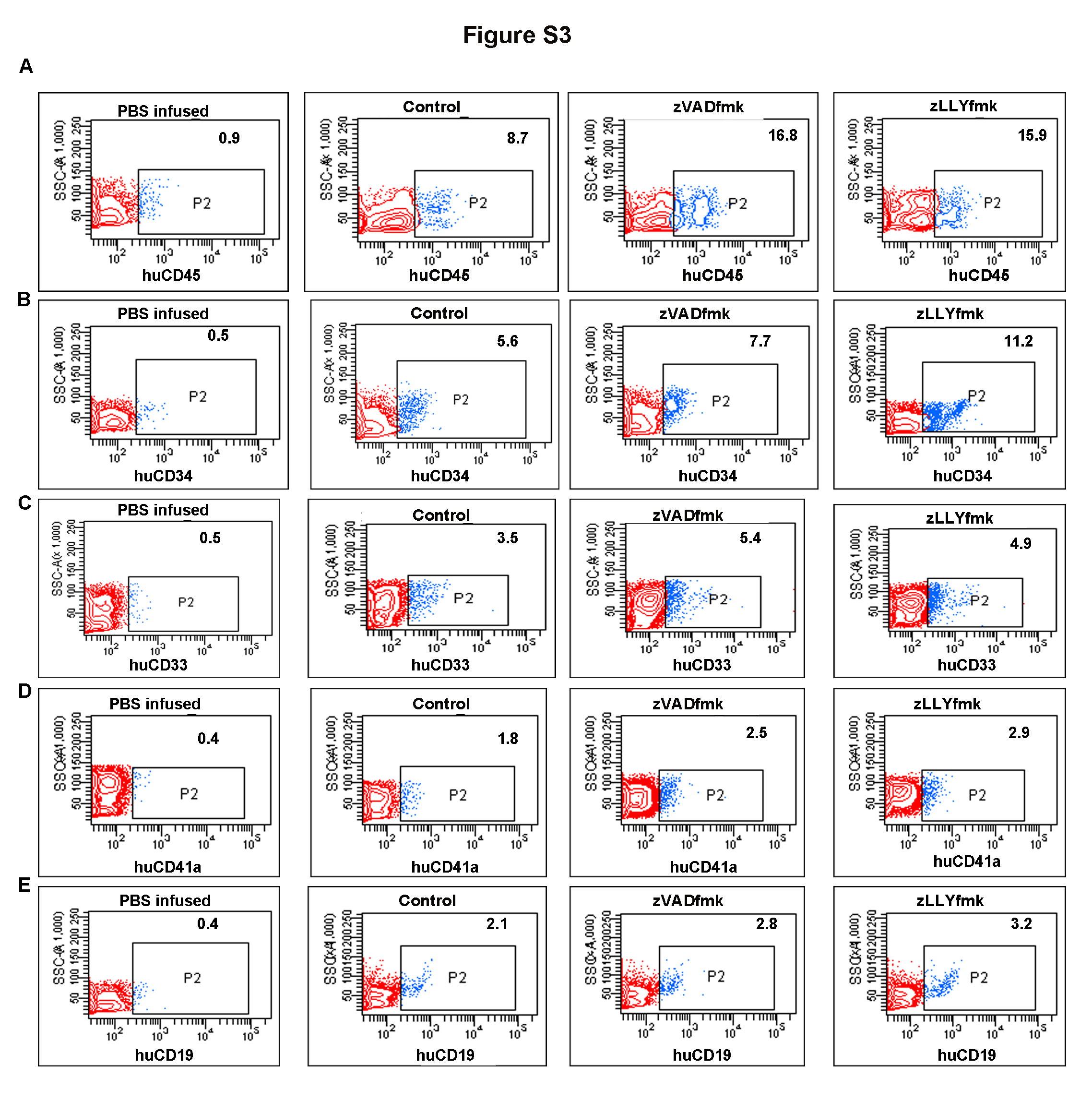

Supplement: Figure S3 — Human multilineage engraftment in the bone marrow of NOD/SCID mice. (A–B) Representative flowcontour plots showing the presence of higher huCD45 and huCD34 engraftment in the marrow of mice that received the cells cultured in the presence of zVADfmk/zLLYfmk (C–E) The human myeloid (CD33), megakaryocyte (CD41a) and B lymphoid (CD19) engraftment in the bone marrow. (1.30 MB TIF) [file pone.0012221.s003.tif]

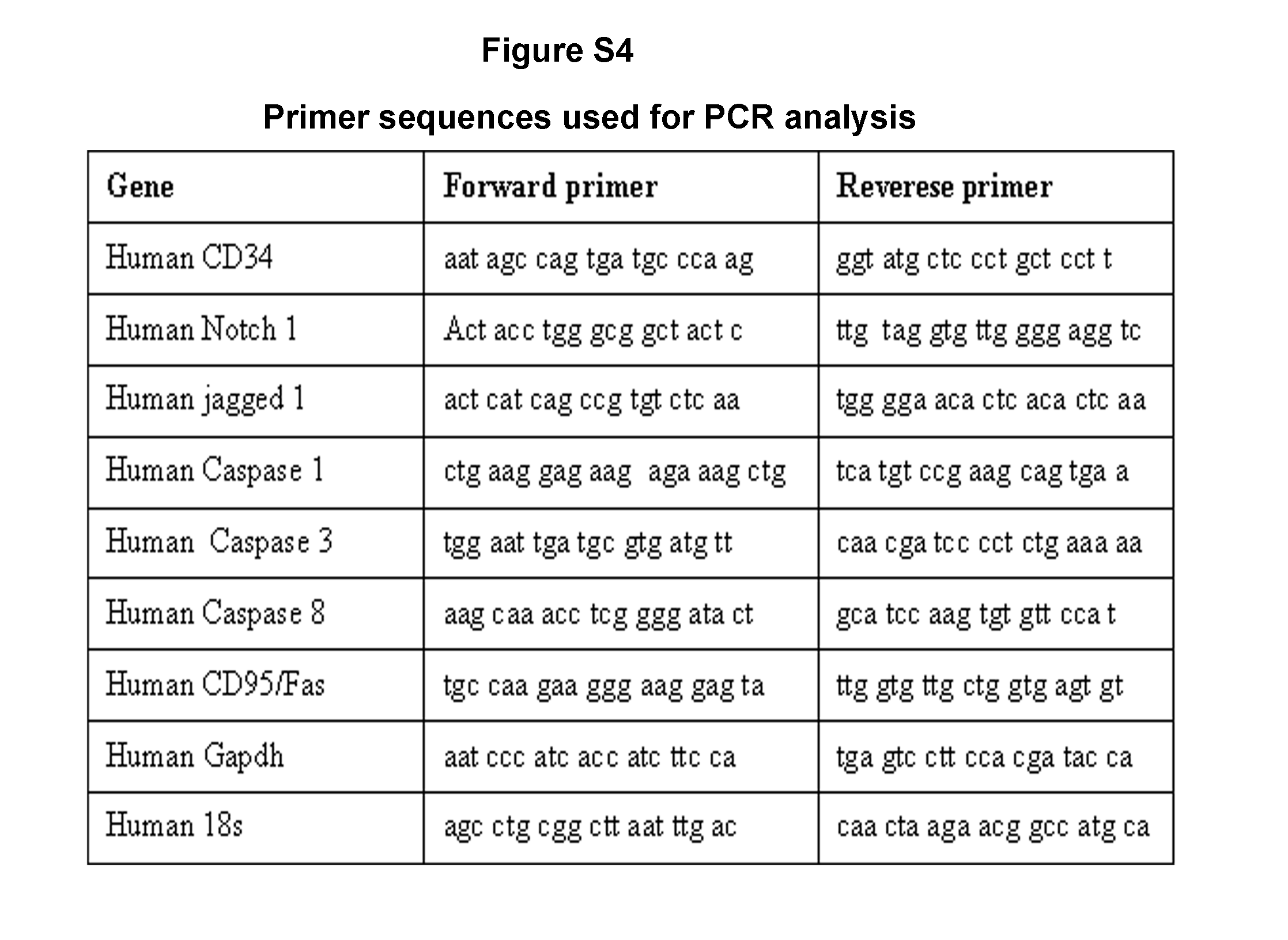

Supplement: Figure S4 — Primer sequences used for PCR analysis. Table summarizes the list of the primer sequences used to perform the PCR reaction. (0.64 MB TIF) [file pone.0012221.s004.tif]
